# Comparison of early clinical outcomes between dual antiplatelet therapy and triple antithrombotic therapy in patients with atrial fibrillation undergoing percutaneous coronary intervention

**DOI:** 10.1371/journal.pone.0264538

**Published:** 2022-02-25

**Authors:** Jiesuck Park, Jin-Hyung Jung, Eue-Keun Choi, Seung-Woo Lee, Soonil Kwon, So-Ryoung Lee, Jeehoon Kang, Kyung-Do Han, Kyung Woo Park, Seil Oh, Gregory Y. H. Lip

**Affiliations:** 1 Department of Internal Medicine, Seoul National University Hospital, Seoul, Republic of Korea; 2 Department of Internal Medicine, Seoul National University College of Medicine, Seoul, Republic of Korea; 3 Department of Medical Statistics, College of Medicine, The Catholic University of Korea, Seoul, Republic of Korea; 4 Department of Statistics and Actuarial Science, Soongsil University, Seoul, Republic of Korea; 5 Liverpool Centre for Cardiovascular Science, University of Liverpool and Liverpool Chest & Heart Hospital, Liverpool, United Kingdom; 6 Department of Clinical Medicine, Aalborg University, Aalborg, Denmark; University of Bologna, ITALY

## Abstract

**Background and objective:**

Most Asian patients with atrial fibrillation (AF) who undergo percutaneous coronary intervention (PCI) receive only dual antiplatelet therapy (DAPT) without oral anticoagulants (vitamin K antagonists [VKA] or non-VKA oral anticoagulants [NOAC]). However, it has not been fully investigated whether the DAPT results in better clinical outcomes in the early period after PCI than the standard triple therapy with VKA or NOAC.

**Methods:**

We analyzed the claims records of 11,039 Korean AF population who had PCI between 2013 and 2018. Patients were categorized according to the post-PCI antithrombotic therapy as VKA-based triple therapy (VKA-TT), NOAC-based triple therapy (NOAC-TT), and DAPT groups. After baseline adjustment using inverse probability weighting, we compared the risks of ischemic endpoints (ischemic stroke, myocardial infarction, and all-cause mortality) and major bleeding at 3 months post-PCI.

**Results:**

Ischemic stroke, MI, and all-cause mortality occurred in 105, 423, and 379 patients, respectively, and 138 patients experienced major bleeding. The DAPT group was associated with a lower risk of ischemic stroke and major bleeding (hazard ratio [HR] 0.55, 95% confidence interval [CI] 0.37–0.84) compared to the VKA-TT group, despite no significant differences in the risks of MI and all-cause mortality. In contrast, the DAPT group demonstrated no significant difference in the risks for ischemic endpoints compared to the NOAC-TT group. Additionally, the DAPT group had a numerically lower risk of major bleeding than the NOAC-TT group but this was not statistically significant (HR 0.69, 95% CI 0.45–1.07).

**Conclusions:**

An outcome benefit of DAPT was observed in the *early period* after PCI compared to the VKA-TT, but not against NOAC-TT users among the Asian AF population. Given the potential long-term benefits of NOACs, greater efforts should be made to increase compliance in clinical practice with proper combination therapy with NOAC after PCI.

## Introduction

Oral anticoagulants (OAC) are essential for stroke prevention in patients with atrial fibrillation (AF) at a moderate-to-high risk of stroke [1]. Approximately 10% of patients with AF require percutaneous coronary intervention (PCI) with coronary stent implantation during the lifetime, where sufficient platelet inhibition is needed to reduce the risk of stent thrombosis [1]. Therefore, in patients with AF who underwent PCI, combination therapy with both OAC and platelet inhibitors is indicated [1, 2]. Previous studies from the vitamin-K antagonist (VKA) era have demonstrated that triple therapy with VKA and dual antiplatelet therapy (DAPT) reduces the risk of ischemic stroke with a cost of higher bleeding events compared to the DAPT only treatment [3, 4]. After the introduction of non-vitamin K OAC (NOAC), the clinical benefit of NOAC with a lower risk of major bleeding than VKA has changed the regimen of post-PCI combination therapy in patients with AF [2]. Previous landmark clinical trials have validated the superiority of NOAC-based combination therapy in bleeding endpoints compared to the conventional triple therapy based on VKA [5–8].

However, in real-world clinical practice, OAC is still underused in the Asian AF population, and the major proportion of those who underwent PCI has received DAPT only without OAC [9, 10]. The high rate of DAPT rather than combination therapy in post-PCI period may represent the preference of the clinicians focusing on the antiplatelet therapy after coronary stent implantation while minimizing the bleeding risk imposed by adding OAC over DAPT [11, 12]. The augmented bleeding risk following combination therapy would be of clinical importance, especially among Asians, given the higher susceptibility for bleeding events related to antithrombotic therapy compared to the Western population [13]. To date, however, there is a paucity of research in the NOAC era providing the real-world data of post-PCI clinical outcomes of DAPT compared to the combination therapy. Therefore, the current nationwide study aimed to compare the 3 months post-PCI ischemic and bleeding risks between DAPT and standard triple therapy with VKA or NOAC among the Asian AF population.

## Materials and methods

### Study population

**Fig 1:**
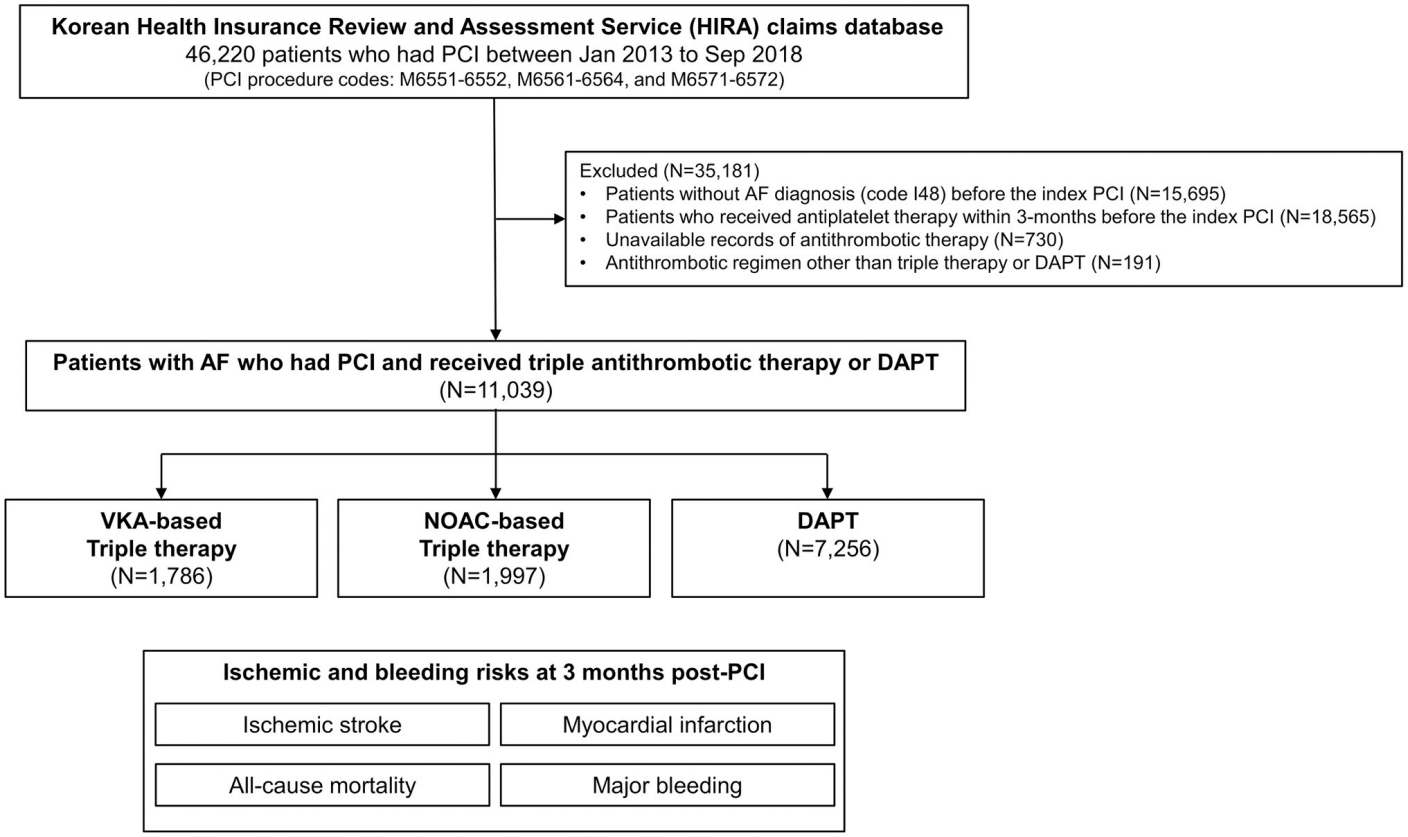
Study flow. Patients who underwent PCI between 2013 and 2018 (N = 46,220) were screened from the HIRA claims database. We excluded patients who were not diagnosed for AF before the index PCI and those who received any platelet inhibitor within 3-month before the index PCI. After additional exclusion of patients with antithrombotic regimens other than triple therapy or DAPT at baseline and those who died before discharge, a total of 11,039 patients were finally included. AF, atrial fibrillation; DAPT, dual antiplatelet therapy; NOAC, non-vitamin K oral anticoagulant; PCI, percutaneous coronary intervention; VKA, vitamin K antagonist.

Patients’ clinical data were obtained from the claims database of the Health Insurance Review and Assessment Service (HIRA) of Korea. The HIRA database includes comprehensive records of healthcare utilization and cost claims obtained from primary care offices, pharmacies, and medical hospitals that cater to the entire Korean population [14]. The data resource can be accessed through the Healthcare Big Data Hub (https://opendata.hira.or.kr) provided by the HIRA database. Since the NOAC had been introduced in Korea as of 2013, we screened patients who underwent PCI between 2013 and 2018 (N = 46,220) (Fig 1). PCI events were identified using the following procedure codes derived from the HIRA database: M6551-6552, M6561-6564, and M6571-6572. Among the eligible patients, we excluded those without AF diagnosis before the index PCI, identified based on the diagnostic codes for AF (I48) from the International Classification of Diseases, Tenth Revision, Clinical Modification (ICD-10-CM). Patients who received antiplatelet therapy within 3 months before the index PCI were excluded, considered chronic antiplatelet users. We investigated the inpatient or outpatient records of OAC (VKA or NOAC) and antiplatelet agent (aspirin, P2Y_12_ inhibitors, including clopidogrel, prasugrel, and ticagrelor) prescribed within 2 weeks after the index PCI (window period). The window period was applied to allow patients to collect their medications from pharmacy and to avoid misclassification for antithrombotic therapy due to delayed claims records for the drug prescriptions. Patients without available records of antithrombotic therapy because of death before discharge or during the window period were excluded, as were patients with unattainable follow-up records. According to the prescription records of the antithrombotic agents, we included patients with triple therapy based on VKA (VKA-TT) or NOAC (NOAC-TT) and those with DAPT (Fig 1). Patients with antithrombotic regimens other than triple therapy or DAPT (N = 191) were excluded. The study design was approved by the Institutional Review Board of the Seoul National University Hospital (E-1911-052-1078). The board committee waived informed consent as all health record data were encrypted for de-identification in the database.

### Clinical risk factors

S1 Table summarizes the detailed definitions of clinical risk factors, which were validated by previous studies [9, 10, 14]. Diabetes mellitus and hypertension were defined based on both diagnostic codes and prescription records of any antihypertensive or antidiabetic drugs, respectively. Congestive heart failure, prior myocardial infarction (MI), peripheral artery disease, stroke or systemic thromboembolism, gastrointestinal bleeding (GIB), intracranial hemorrhage (ICH), and chronic liver or renal disease were defined based on the International Classification of Diseases, Tenth Revision, Clinical Modification codes. CHA_2_DS_2_-VASc scores were calculated for each patient to estimate the individual risk of stroke. Data for the labile international normalized ratio and alcohol consumption are not available in the HIRA database; therefore, we defined modified HAS-BLED scores [9] by excluding these variables to determine the individual risk of bleeding.

### Study outcomes

Patients were followed up from at the end of the window period until the event date of clinical outcomes or the end of the study period of 3 months, whichever comes first. The ischemic endpoints included ischemic stroke, MI, and all-cause mortality. The bleeding endpoint was major bleeding from vital organs, including GIB or ICH. S1 Table shows definitions of each clinical outcome. In brief, ischemic stroke was defined as inpatient primary diagnostic codes combined with claims for brain imaging studies, including computed tomography or magnetic resonance imaging. MI and major bleeding were defined based on inpatient primary diagnostic codes.

### Statistical analysis

We performed an intergroup comparison of the ischemic and bleeding risks at 1-, 2-, and 3-months periods. Inverse probability weighting (IPW) was applied to adjust baseline characteristics between the study groups using stabilized weights calculated from the propensity score [15]. The propensity score was calculated by the multinomial logistic regression model using the baseline characteristics: demographics (age and sex); comorbidities (diabetes mellitus, hypertension, dyslipidemia, congestive heart failure, prior MI, prior PCI or bypass surgery, peripheral artery disease, prior stroke or ICH, prior GIB, chronic renal and liver disease, and prior OAC treatment); and concomitant medications (non-steroidal anti-inflammatory drugs, statins, loop diuretics, beta-blockers, calcium channel blockers, renin-angiotensin-aldosterone system blockers, and proton pump inhibitors). The balance between the groups after IPW was assessed by the absolute standardized difference (ASD). We considered the maximum ASD <0.1 (10%) as a negligible level of differences [16]. The incidence rates were calculated based on the event numbers of the clinical outcomes over 3 months among the weighted population. We employed weighted Kaplan-Meier curves to plot the time-to-event distribution, and differences in the event-free rate were assessed by the log-rank test. Additionally, the weighted Cox hazard regression model was used to estimate hazard ratios (HR) for the clinical outcomes. Prespecified subgroup analyses were performed according to age (≤65 or >65 years), prior OAC treatment, and after patient stratification according to their baseline stroke (CHA_2_DS_2_-VASc score ≤4 or >4) and bleeding (modified HAS-BLED score ≤3 or >3) risks. In addition, we evaluated whether the DAPT group with potent P2Y_12_ inhibitors (prasugrel or ticagrelor) was associated with a lower ischemic risk compared to the triple therapy groups. All statistical analyses were performed using the R software, version 3.4.3 (R Development Core Team, Vienna, Austria). All probability values were 2-sided, with a p-value <0.05 considered statistically significant.

## Results

### Baseline characteristics

Before IPW, patients in the NOAC-TT groups were older, had higher likelihood of heart failure, and had higher CHA_2_DS_2_-VASc score than the other groups (S2 Table). In contrast, the prevalence of previous MI was highest in the DAPT group. The intergroup differences in the baseline characteristics were well-balanced after IPW, resulting in a weighted number of 1,734, 1,947, and 7,374 patients for the VKA-TT, NOAC-TT, and DAPT group, respectively (Table 1). The median (interquartile range [IQR]) age of the weighted population was 72 (63–78), and the median (IQR) CHA_2_DS_2_-VASc and HAS-BLED scores were 4 (2–5) and 3 (3–4), respectively. In the weighted population, 25% of the VKA-TT group received VKA previously, whereas 24% of the NOAC-TT group had prior NOAC. In the DAPT group, 28% of patients received potent P2Y_12_ inhibitors.

**Table 1:** Baseline characteristics of IPW population.

Groups	VKA-based TT	NOAC-based TT	DAPT	ASD
Number of weighted patients	1,734	1,947	7,374	
*Demographics*				
Age, years	72 (63–78)	72 (63–78)	72 (62–78)	0.051
Age, groups				0.013
<65	509 (29.4)	577 (29.7)	2,207 (29.9)	
65–74	497 (28.6)	573 (29.4)	2,198 (29.8)	
75≤	729 (42.0)	797 (40.9)	2,969 (40.3)	
Women	612 (35.3)	680 (34.9)	2,517 (34.1)	0.025
*Comorbidities*				
Diabetes mellitus	626 (36.1)	714 (36.7)	2,668 (36.2)	0.012
Hypertension	1,498 (86.4)	1,666 (85.5)	6,244 (84.7)	0.048
Dyslipidemia	1,466 (84.5)	1,626 (83.5)	6,193 (84.0)	0.028
Congestive heart failure	759 (43.8)	865 (44.4)	3,206 (43.5)	0.019
Prior MI	847 (48.8)	958 (49.2)	3,583 (48.6)	0.012
Prior PCI	94 (5.4)	99 (5.1)	410 (5.6)	0.021
Prior CABG	6 (0.4)	0 (0.0)	14 (0.2)	0.084
Peripheral artery disease	428 (24.7)	454 (23.3)	1,760 (23.9)	0.033
Prior stroke / TIA / STE	263(15.2)	284 (14.6)	1,100 (14.9)	0.016
Prior ICH	9 (0.5)	17 (0.9)	53 (0.7)	0.046
Prior GI bleeding	123 (7.1)	132 (6.8)	504 (6.8)	0.012
Renal disease	299 (17.2)	347 (17.8)	1,259 (17.1)	0.019
Liver disease	652 (37.6)	718 (36.8)	2,710 (36.8)	0.017
Prior OAC user^a^	467 (26.9)	521 (26.8)	2,016 (27.3)	0.013
Prior warfarin user	428 (24.6)	58 (3.0)	1,005 (13.6)	
Prior NOAC user	39 (2.3)	463 (23.8)	1,011 (13.7)	
*CHA*_*2*_ *DS*_*2*_ *-VASc score*				
median (IQR)	4 (2–5)	4 (2–5)	4 (2–5)	0.066
0	19 (1.1)	22 (1.1)	131 (1.8)	
1	142 (8.2)	156 (8.0)	731 (9.9)	
2	321 (18.5)	353 (18.1)	1,339 (18.2)	
3	343 (19.8)	418 (1.5)	1,398 (19.0)	
4	288 (16.6)	347 (17.8)	1,303 (17.7)	
5	309 (17.8)	300 (15.4)	1,106 (15.0)	
6	180 (10.4)	213 (10.9)	776 (10.5)	
7≤	134 (7.7)	138 (7.1)	590 (8.0)	
*Modified HAS-BLED*				
median (IQR)	3 (3–4)	3 (3–4)	3 (3–4)	0.061
1	76 (4.4)	63 (3.2)	321 (4.4)	
2	284 (16.4)	374 (19.2)	1,358 (18.4)	
3	729 (42.0)	801 (41.1)	2,955 (40.1)	
4	429 (24.8)	488 (25.1)	1,966 (26.7)	
5	190 (10.9)	179 (9.2)	665 (9.0)	
6≤	27 (1.5)	43 (2.2)	110 (1.5)	
*Concomitant medication*				
Clopidogrel	1,637 (94.4)	1,795 (92.2)	5,285 (71.7)	
Prasugrel or Ticagrelor	97 (5.6)	152 (7.8)	2,089 (28.3)	
NSAIDs	1,153 (66.5)	1,266 (65.0)	4,828 (65.5)	0.030
Statins	1,571 (90.6)	1,731 (88.9)	6,591 (89.4)	0.055
Loop diuretics	1,063 (61.3)	1,121 (57.6)	4,262 (57.8)	0.075
Beta-blockers	1,450 (83.6)	1,565 (80.4)	6,011 (81.5)	0.084
Calcium channel blockers	1,162 (67.0)	1,280 (65.7)	4,896 (66.4)	0.027
RAAS blockers	1,390 (80.1)	1,528 (78.5)	5,744 (77.9)	0.055
Proton pump inhibitors	1,010 (58.2)	1,147 (58.9)	4,295 (58.2)	0.013

^a^within 1-year before the index PCI

ASD, absolute standardized difference; CABG, coronary artery bypass grafting; DAPT, dual-antiplatelet therapy; GI, gastrointestinal; ICH, intracranial hemorrhage; IPW, inverse probability weighting; IQR, inter-quartile range; MI, myocardial infarction; NOAC, non-vitamin K oral anticoagulant; NSAIDs, non-steroidal anti-inflammatory drugs; OAC, oral anticoagulant; PCI, percutaneous coronary intervention; RAAS, renin-angiotensin-aldosterone system; SD, standard deviation; STE, systemic thromboembolism; TIA, transient ischemic attack; TT, triple therapy; VKA, vitamin K antagonist.

Values given as median (interquartile range), or number (percentage), unless otherwise indicated.

### Three months post-PCI ischemic and bleeding risks of DAPT vs. triple therapy

**Fig 2:**
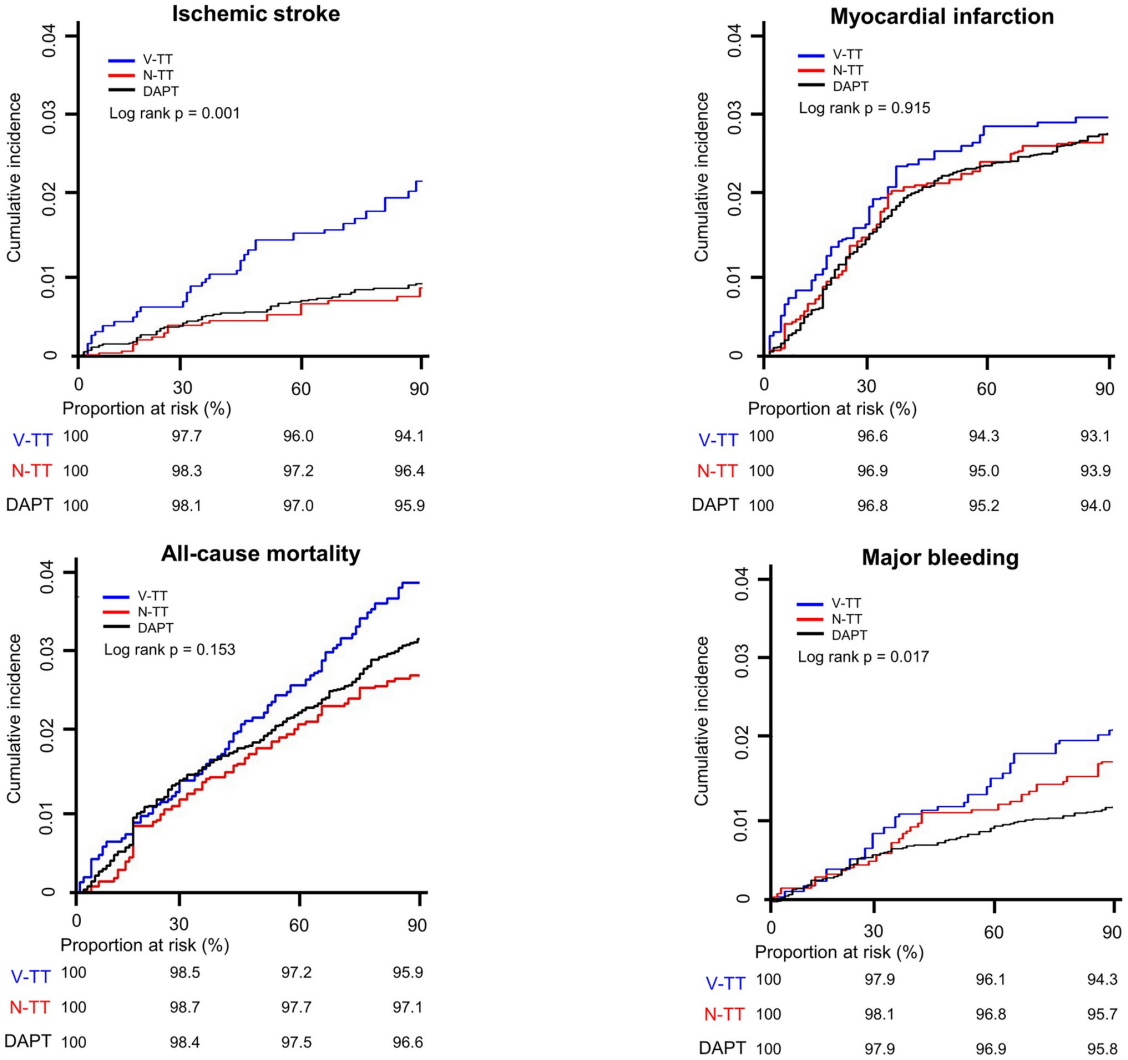
Weighted Kaplan-Meier curves for clinical outcomes according to antithrombotic therapy. The incidence of ischemic stroke was higher in the VKA-TT than in the NOAC-TT and DAPT groups. However, there was no difference in the incidence of all-cause mortality and non-fatal MI between the DAPT and triple therapy groups. In terms of the bleeding, the DAPT group showed a lower incidence of major bleeding than the triple therapy groups. DAPT, dual antiplatelet therapy; NOAC, non-vitamin K oral anticoagulant; N-TT, NOAC-based triple therapy; VKA, vitamin K antagonist; V-TT, VKA-based triple therapy.

**Fig 3:**
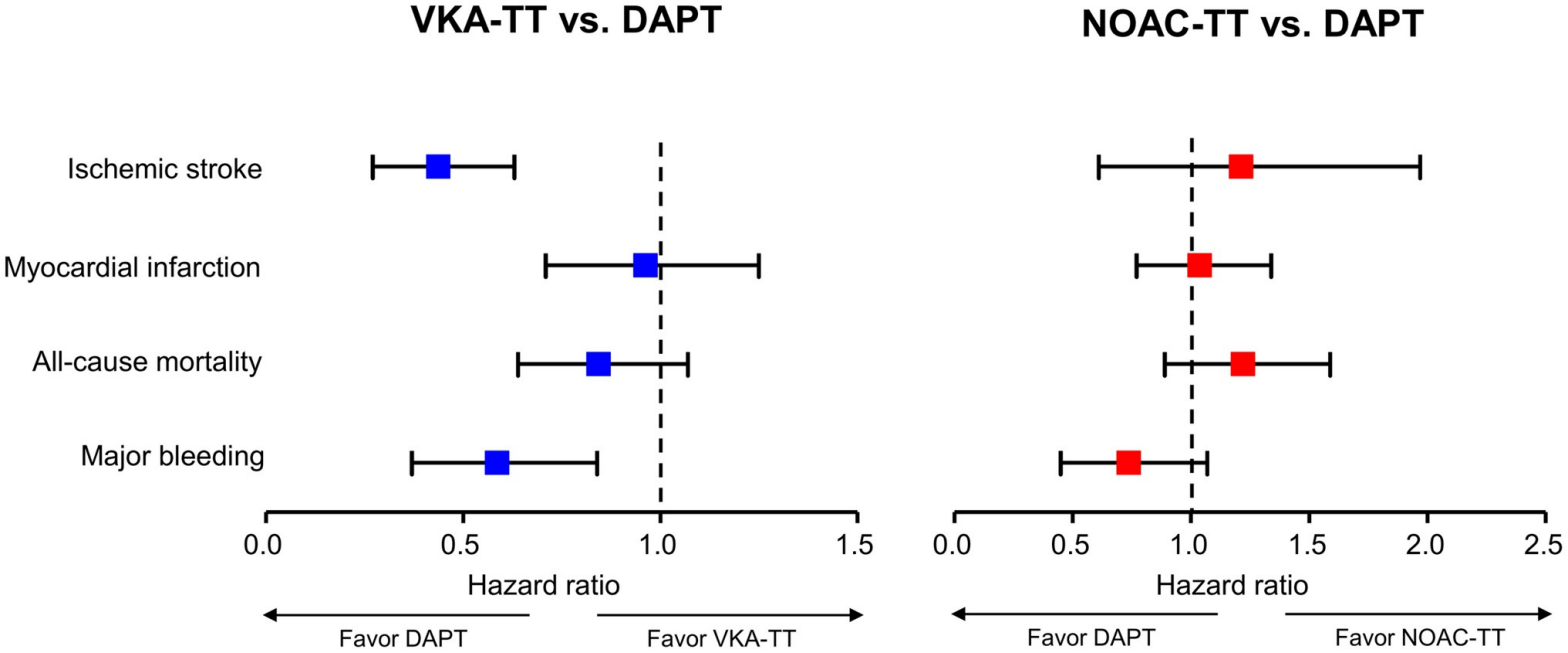
Hazard ratios for ischemic and bleeding risks at 3 months according to antithrombotic therapy. The forest plots represent the ischemic and bleeding risks at 3 months post-PCI of the DAPT compared to the triple therapy based on VKA (left panel) or NOAC (right panel). The DAPT group showed a lower risk of ischemic stroke and major bleeding than the VKA-TT group. In contrast, the DAPT group demonstrated no significant differences in the risks of ischemic and bleeding endpoints compared to the NOAC-TT group. CI, confidence interval; DAPT, dual antiplatelet therapy; NOAC, non-vitamin K oral anticoagulants; PCI, percutaneous coronary intervention; TT, triple therapy; VKA, vitamin K antagonists.

During the 3 months, ischemic stroke, MI, and all-cause mortality occurred in 105, 423, and 379 patients, respectively, and 138 patients experienced major bleeding. The DAPT group was associated with a lower risk of ischemic stroke compared to the VKA-TT group (incidence rate [IR] 3.2 vs. 7.9 per 100 person-year; HR 0.41, 95% CI 0.27–0.63) (Figs 2 and 3, and Table 2). In contrast, there were no significant differences in the risks of MI and all-cause mortality between the DAPT and VKA-TT groups. For the bleeding endpoint, the DAPT group was associated with a lower risk of major bleeding than the VKA-TT group (IR 4.3 vs. 7.8 per 100 person-year; HR 0.55, 95% CI 0.37–0.84). On the contrary, the DAPT group demonstrated no significant differences in the risks for ischemic endpoints compared to the NOAC-TT group (Figs 2 and 3, and Table 2). In addition, the DAPT group showed no significant differences for the major bleeding compared to the NOAC-TT group (IR 4.3 vs. 6.3 per 100 person-year; HR 0.69, 95% CI 0.45–1.07).

**Table 2:** Comparison of clinical outcomes according to antithrombotic therapy.

Outcomes	30 Days Outcome					60 Days Outcome				90 Days Outcome			
	Group	Event	IR*	HR† (95% CI)	HR† (95% CI)	Event	IR*	HR† (95% CI)	HR† (95% CI)	Event	IR*	HR† (95% CI)	HR† (95% CI)
Ischemic stroke	V-TT	14	9.4	1 (reference)		24	8.3	1 (reference)		34	7.9	1 (reference)	
	N-TT	6	4.0	0.43 (0.16–1.06)	1 (reference)	11	3.5	0.42 (0.20–0.83)	1 (reference)	14	3.0	0.38 (0.20–0.70)	1 (reference)
	DAPT	27	4.6	0.49 (0.26–0.97)	1.15 (0.52–2.97)	44	3.8	0.45 (0.28–0.76)	1.08 (0.58–2.20)	57	3.2	0.41 (0.27–0.63)	1.06 (0.61–1.97)
Myocardial infarction	V-TT	53	37.2	1 (reference)		59	21.0	1 (reference)		61	14.6	1 (reference)	
	N-TT	60	37.8	0.91 (0.59–1.41)	1 (reference)	74	23.6	0.85 (0.58–1.22)	1 (reference)	79	17.1	0.93 (0.65–1.32)	1 (reference)
	DAPT	226	38.7	0.85 (0.60–1.22)	0.93 (0.66–1.33)	258	22.4	0.84 (0.63–1.14)	1.00 (0.74–1.35)	283	16.5	0.93 (0.71–1.25)	1.00 (0.77–1.34)
All-cause mortality	V-TT	27	18.5	1 (reference)		51	17.5	1 (reference)		74	17.2	1(Ref.)	
	N-TT	26	16.3	0.88 (0.51–1.51)	1 (reference)	45	14.1	0.80 (0.54–1.20)	1 (reference)	57	11.9	0.70 (0.49–0.98)	1 (reference)
	DAPT	115	19.5	1.05 (0.70–1.63)	1.20 (0.80–1.87)	179	15.2	0.87 (0.64–1.20)	1.08 (0.79–1.52)	248	14.1	0.82 (0.64–1.07)	1.18 (0.89–1.59)
Major Bleeding	V-TT	14	10.0	1 (reference)		24	8.3	1 (reference)		33	7.8	1 (reference)	
	N-TT	11	6.6	0.66 (0.29–1.46)	1 (reference)	21	6.5	0.79 (0.44–1.43)	1 (reference)	30	6.3	0.80 (0.49–1.32)	1 (reference)
	DAPT	40	6.7	0.67 (0.38–1.27)	1.02 (0.54–2.11)	60	5.1	0.62 (0.39–1.01)	0.78 (0.48–1.32)	75	4.3	0.55 (0.37–0.84)	0.69 (0.45–1.07)

* Incidence rate per 100 person-year.

^†^ HR were estimated after multivariable adjustment. See Methods.

CI, confidence interval; DAPT, dual-antiplatelet therapy; HR, hazard ratios; IR, incidence rate; IPW, inverse probability weighting; N-TT, NOAC-based triple therapy; V-TT, VKA-based triple therapy.

### Subgroup analysis

**Fig 4:**
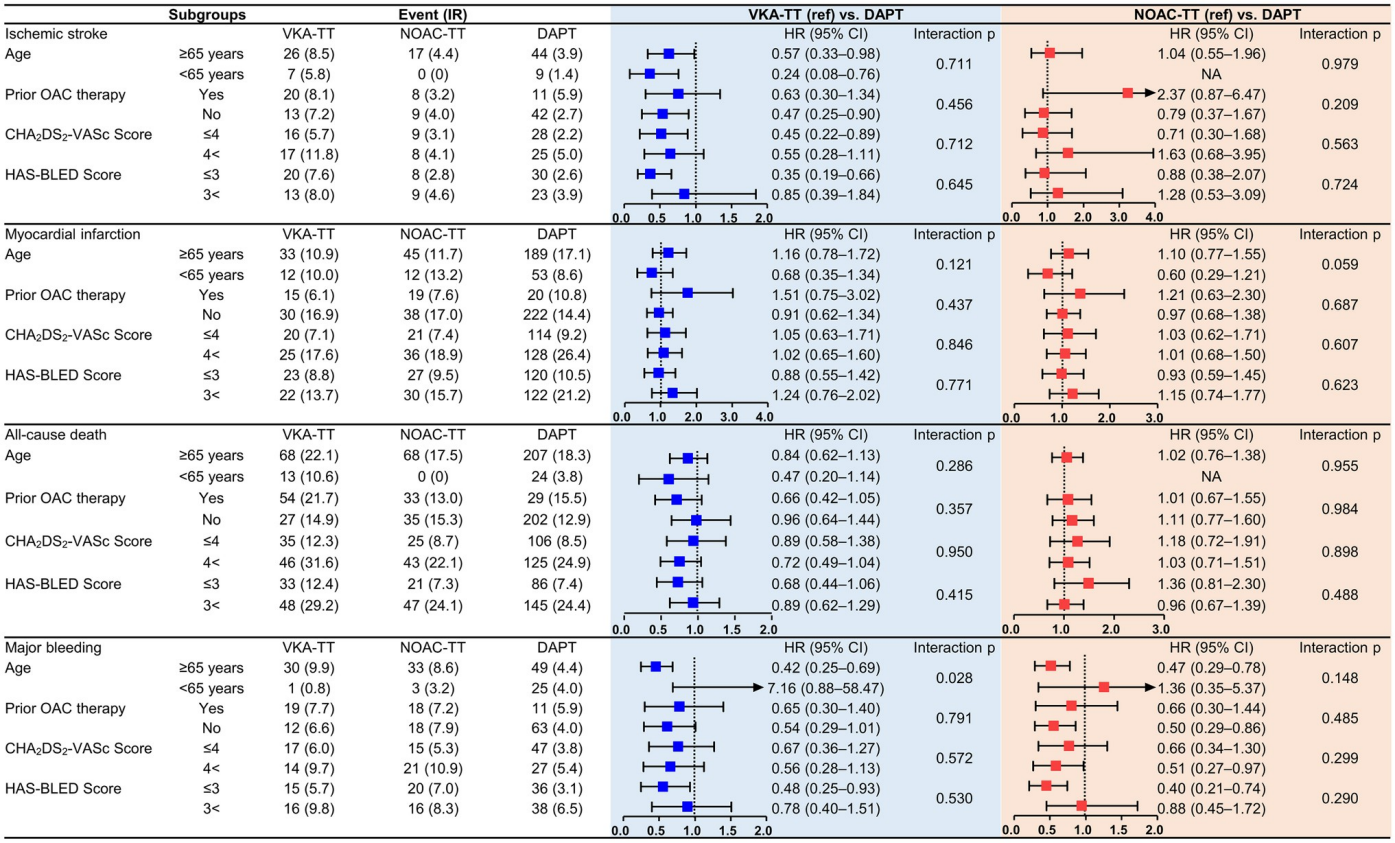
Subgroup analysis for clinical outcomes according to antithrombotic therapy. Prespecified subgroup analysis was performed in elderly patients (age >65 years), those with potent P2Y_12_ inhibitors (prasugrel or ticagrelor), according to prior OAC treatment, and after patient stratification according to their baseline stroke and bleeding risks. The incidence rate was expressed as the number of events per 100-person year. The HR was estimated after the adjustment for baseline characteristics. CI, confidence interval; DAPT, dual antiplatelet therapy; HR, hazard ratios; IR, incidence rate; NOAC-TT, non-vitamin K oral anticoagulant-based triple therapy; OAC, oral anticoagulants; VKA-TT, vitamin K antagonist-based triple therapy.

The DAPT group showed a consistent trend across the subgroups with lower risks of ischemic stroke and major bleeding compared to the VKA-TT group (Fig 4). The higher risk of major bleeding with VKA-TT was prominent in the elderly patients (p for interaction = 0.028). In contrast, no significant differences were observed across the subgroups for the ischemic endpoints between the DAPT and the NOAC-TT group. In patients with old age, those without prior OAC treatment, and those with low bleeding tendency, the DAPT group showed a lower trend of major bleeding than the NOAC-TT group despite no significant interactions. The DAPT group with potent P2Y_12_ inhibitors showed no significant differences in the risks of ischemic and bleeding endpoints compared to the triple therapy groups (S3 Table).

## Discussion

The current nationwide study evaluated the ischemic and bleeding risks at 3 months post-PCI with DAPT vs. triple therapy among the Asian AF population. DAPT group was associated with lower risks of ischemic stroke and major bleeding than the VKA-TT group, but no significant differences were observed for MI and all-cause mortality. In contrast, the DAPT group demonstrated no significant differences for ischemic and bleeding endpoints compared to the NOAC-TT group.

Indications for both OAC and antiplatelet therapy in patients with AF who underwent PCI have resulted in a concept of combination antithrombotic therapy [1, 2]. However, excessive bleeding risk by combining these two classes has been a long-lasting concern in clinical practice [11], especially among Asians with a higher susceptibility to OAC-induced bleeding compared to Westerns [13]. Poor compliance to the treatment guidelines is also prevalent among the Asian AF population, with a low rate of post-PCI OAC but higher use of DAPT [9, 10]. Previous studies have found that increased bleeding tendency, but also concomitant cardiovascular disease (MI or peripheral vasculopathy) are associated with low compliance to combination treatment after PCI [9, 10]. These findings may reflect a preference of DAPT among the clinicians, focusing on platelet inhibition early after coronary stenting while mitigating augmented bleeding risk by adding OAC [11, 12].

Previous meta-analysis conducted in the VKA era had shown that post-PCI DAPT was associated with lower bleeding risk than VKA-TT but exhibited no benefit in the risks of MI and major cardiovascular events [3, 4]. Our results are similar to previous findings demonstrating no significant differences in MI and all-cause mortality between the DAPT and triple therapy groups. However, we observed that the risk of ischemic stroke at 3 months was higher in the VKA-TT than in the DAPT group. The cumulative incidence of the ischemic stroke shows an early deviation between the VKA-TT and DAPT groups, with consistent divergence throughout the follow-up period. This result would have been contributed by prevailing suboptimal dosing of VKA for stroke prevention among the Asian AF population [17, 18]. The risk of major bleeding in the VKA-TT group notably increases from the 1-month post-PCI period, which may further discourage clinicians from maintaining sufficient antithrombotic therapy, paradoxically leading to a higher rate of ischemic stroke.

Recent landmark clinical trials have demonstrated the safety benefit of combination therapy with NOAC compared to the VKA-TT [19]. However, the post-PCI use of combination therapy remains low among the Asian AF population even after the introduction of NOAC, despite the increasing use of the latter in recent times [9]. To date, it has not been investigated whether the use of DAPT results in better clinical outcomes in the early period after PCI compared to NOAC-based combination therapy. In our results, the DAPT group showed no significant differences in the ischemic and bleeding outcomes compared to the NOAC-TT group. This finding counters the underlying preference among the clinicians with the higher rate of DAPT, focusing on the platelet inhibition after coronary stent implantation while avoiding potential bleeding risks with combination therapy [11, 12]. Rather, the antithrombotic effects of NOAC in combination with DAPT may further reduce the subsequent (long-term) risks of thromboembolic and cardiovascular events, with appropriate mitigation strategies for bleeding [2].

In subgroup analysis, the lower trend of major bleeding in the DAPT group than the triple therapy groups was notable in patients with old age, those without prior OAC treatment, and those with low bleeding tendency. Old age is one of the major risk factors for the bleeding event in AF [20]. The yearly trend in the clinal characteristics of the Asian AF population undergoing PCI indicates an increasing mean age of the patients [9]. This would translate into a growing burden for the clinicians adhering to the combination antithrombotic therapy post-PCI among elderly patients. The bleeding risk following the combination treatment would also be differed according to prior OAC therapy [21], where patients on chronic OAC would be more stable to post-PCI combination therapy than those who newly administered multiple antithrombotic agents [22]. The incidence of major bleeding was higher in all study groups with higher bleeding tendency at baseline. However, the difference in the bleeding incidence between the DAPT and the triple therapy groups was prominent in patients with low bleeding tendency at baseline (modified HAS-BLED score ≤3). The addition of OAC over DAPT, per se, promotes the bleeding event in patients with AF [23]. Therefore, strategies to reduce the potential risk of post-PCI bleeding would be essential such as minimal period of triple therapy, coronary intervention via radial access, or gastric protection with proton pump inhibitors.

Given the high susceptibility to OAC-induced bleeding among the Asians [13], one may prefer to focus on platelet inhibition with DAPT initially after PCI without OAC [12]. Our findings on the non-significant differences in 3-month outcomes between the DAPT and NOAC-TT group may in part suggest short-term DAPT as a treatment option for the Asian AF population at high bleeding risk. However, the efficacy and safety of post-PCI DAPT in the AF population need to be validated in future clinical trials with a longer time frame. The upcoming WOEST 3 (What is the Optimal Antithrombotic Strategy in Patients Presenting With Acute Coronary Syndrome Having Atrial Fibrillation With Indication for Anticoagulants?) trial (NCT04436978) [24] is expected to assess the 1-year bleeding and thromboembolic complications of the modified combination therapy with first-month DAPT compared to the standard treatment.

We observed no detectable difference in the ischemic risk between the triple therapy (mainly with clopidogrel) and the DAPT group with potent P2Y_12_ inhibitors. The incremental effect of platelet inhibition by combining OAC over DAPT might be comparable to that by switching clopidogrel to more potent P2Y_12_ inhibitors. Previous nationwide study among the Asian population with acute coronary syndrome has noted an increased bleeding risk with the potent P2Y_12_ inhibitors compared to clopidogrel [25]. The higher susceptibility among the Asians to bleeding events with potent P2Y_12_ inhibitors may contribute to the comparable risk of major bleeding observed between the triple therapy and DAPT group with potent P2Y_12_ inhibitors [13].

Our findings should be interpreted under the following limitations. Because of the non-randomized study design, possible crossovers or premature discontinuation of the antithrombotic therapy could affect our results. To overcome this limitation, we compared the early clinical outcomes at 3 months post-PCI to minimize these variabilities. Additionally, the post-PCI rates of triple therapy and DAPT were similar between the baseline and 3 months periods among the Korean AF population, reducing the possibilities of temporal variation in both treatment groups [26]. However, because of the relatively short follow-up period, the current study is limited to provide long-term clinical outcomes associated with different antithrombotic therapy. We infer that the higher risk of the ischemic stroke observed in the VKA-TT than in the DAPT group might be contributed by the suboptimal dosing of VKA. However, as laboratory data are not available in the HIRA database, we could not provide time in therapeutic range of the VKA in the VKA-TT group. Therefore, the current findings on the VKA-TT group need to be interpreted with caution and not to be considered as causal. Even though we corrected the differences in the baseline characteristics with IPW, unmeasured confounders may exist, affecting the study results. Finally, the data of PCI procedures and the coronary lesion characteristics are not included in the current study as this information are not available in the HIRA database.

## Conclusions

Among the Asian AF population, post-PCI DAPT without OAC was more prevalent than combination antithrombotic therapy even in the NOAC era. An outcome benefit of DAPT was observed in the early period after PCI compared to the VKA-TT. However, DAPT showed no statistically significant benefit in the ischemic and bleeding outcomes compared to the NOAC-TT. Given the potential long-term benefits of NOACs, greater efforts should be made to increase compliance in the clinical practice with proper combination therapy with NOAC after PCI.

## Supporting information

S1 Table Definition of comorbidity / scores / outcomes. (PDF)

S2 Table Baseline characteristics of crude population. (PDF)

S3 Table Clinical outcomes in dual-antiplatelet therapy group with potent P2Y_12_ inhibitor. (PDF)
