# Supplementary material for: Comparison of early clinical outcomes between dual antiplatelet therapy and triple antithrombotic therapy in patients with atrial fibrillation undergoing percutaneous coronary intervention
Source: PLoS One. 2022 Feb 25;17(2):e0264538. doi: 10.1371/journal.pone.0264538 (PMC8880831; doi:10.1371/journal.pone.0264538)
Supplement: S1 Table — (PDF) [file pone.0264538.s001.pdf]

S1 Table. Definition of comorbidity / scores / outcomes

Diagnosis	ICD-10-CM code and definition	Diagnostic definition
**Inclusion criteria**		
Atrial fibrillation	I48.0-48.4, I48.9	Admission or outpatient department≥1
Percutaneous coronary intervention	M6551-6552, M6561-6564, M6571-6572	Admission and procedure codes
**Comorbidities**		
Hypertension	I10-I13, I15; and minimum 1 prescription of anti-hypertensive drug (thiazide, loop diuretics, aldosterone antagonist, alpha-/beta-blocker, calcium-channel blocker, angiotensin-converting enzyme inhibitor, angiotensin II receptor blocker).	Admission≥1 or outpatient department≥2
Diabetes mellitus	E11-E14; and minimum 1 prescription of anti-diabetic drugs (sulfonylureas, metformin, meglitinides, thiazolidinediones, dipeptidyl peptidase-4 inhibitors, α-glucosidase inhibitors and insulin).	Admission≥1 or outpatient department≥2
Dyslipidemia	E78	Admission or outpatient department≥1
Congestive heart failure	I50	Admission or outpatient department≥1
Prior Stroke	I63, I64	Admission or outpatient department≥1
Transient ischemic attack	G458, G459	Admission or outpatient department≥1
Systemic thromboembolism	I26, I74, I802	Admission or outpatient department≥1
Prior intracranial hemorrhage	I60-I62	Admission or outpatient department≥1
Prior myocardial infarction	I21, I22	Admission or outpatient department≥2
Peripheral artery disease	I70, I73	Admission or outpatient department≥1
Renal disease	I13.1, N03, N05, N10-N19, Z49, Z94.0, Z99.2	Admission or outpatient department≥1
Liver disease	K70, K72-K76, K71.3-K71.7	Admission or outpatient department≥1
Prior PCI	M6551-6552, M6561-6564, M6571-6572	Procedure codes
Prior CABG	O1640, O1641, O1647, O1648, O1649, OA640, OA641, OA647, OA648, OA649	Procedure codes
**Scores**		

CHA_2DS_2-VASc score	Heart failure (1 point), hypertension (1 point), age ≥75 years (2 points), diabetes (1 point), previous stroke/systemic embolism/transient ischemic attack (2 points), vascular disease (prior MI or PAD, 1 point) and female sex (1 point)	
Modifed HAS-BLED score*	Hypertension (1 point), liver disease (1 point), renal disease (1 point), stroke history (1 point), bleeding history (1 point), age>65 years (1 point) and drug (concomitant use of NSAID or antiplatelet agent, 1 point)	
**Outcomes**		
Non-fatal myocardial infarction	I21, I22	Primary diagnosis, admission ≥1
Ischemic stroke	I63, I64	Primary diagnosis, admission≥1 (≥3 days) and brain imaging (CT or MRI) ≥1
Major bleeding	Intracranial hemorrhage or gastrointestinal bleeding or extracranial/unclassified major bleeding	Each definition was described as below.
Gastrointestinal bleeding	I85, K22.1, I22.8, K25.0, K25.2, K25.4, K25.6, K26.0, K26.2, K26.4, K26.6, K27.0, K27.2, K27.4, K27.6, K28.0, K28.2, K28.4, K28.6, K29.0, K31.8, K92.0, K92.1, K92.2, K55.2, K57.0, K57.1, K57.2, K57.3, K57.4, K57.5, K57.8, K57.9, K62.5, K66.1	Primary diagnosis, admission ≥1
Intracranial hemorrhage	I60-62	Primary diagnosis, admission≥1 (≥3 days) and brain imaging (CT or MRI) ≥1
Extracranial/unclassified major bleeding	D62, H05.2, H35.6, H43.1, J94.2, M25.0, R04	Primary diagnosis and [admission≥1 or RBC transfusion]

* Labile international normalized ratio (INR) and alcohol use could not be evaluated from claims and were excluded from scoring in this study.
CABG, coronary artery bypass grafting; PCI, percutaneous coronary intervention.
