# Supplementary material for: Comparison of early clinical outcomes between dual antiplatelet therapy and triple antithrombotic therapy in patients with atrial fibrillation undergoing percutaneous coronary intervention
Source: PLoS One. 2022 Feb 25;17(2):e0264538. doi: 10.1371/journal.pone.0264538 (PMC8880831; doi:10.1371/journal.pone.0264538)
Supplement: S2 Table — (PDF) [file pone.0264538.s002.pdf]

S2 Table. Baseline characteristics of crude population

Groups	(A) VKA-based TT	(B) NOAC-based TT	(C) DAPT	p-value A vs. B	p-value A vs. C	p-value B vs. C
Number of patients	1,786	1,997	7,256			
Demographics						
Age, years	72 (63 - 77)	74 (67 - 79)	71 (60 - 78)	<0.001	0.002	<0.001
Age, groups				<0.001	<0.001	<0.001
<65	503 (28.2)	378 (18.9)	2,558 (35.3)			
65-74	614 (34.4)	631 (31.6)	2,052 (28.3)			
75≤	669 (37.5)	988 (49.5)	2,646 (36.5)			
Women	602 (33.7)	706 (35.4)	2,417 (33.3)	0.288	0.750	0.087
Comorbidities						
Diabetes mellitus	712 (39.9)	733 (36.7)	2,519 (34.7)	0.456	<0.001	0.099
Hypertension	1,560 (87.4)	1,762 (88.2)	5,970 (82.3)	0.405	<0.001	<0.001
Dyslipidemia	1,456 (81.5)	1,685 (84.4)	6,114 (84.3)	0.020	0.005	0.900
Congestive heart failure	899 (50.3)	1,125 (56.3)	2,695 (37.1)	<0.001	<0.001	<0.001
Prior MI	725 (40.6)	764 (38.3)	3,864 (53.3)	0.142	<0.001	<0.001
Prior PCI	79 (4.4)	91 (4.6)	463 (6.4)	0.843	0.002	0.002
Prior CABG	4 (0.2)	0 (0.0)	14 (0.2)	0.034	0.792	0.050
Peripheral artery disease	363 (20.3)	495 (24.8)	1,742 (24.0)	0.001	0.001	0.471
Prior stroke / TIA / STE	321 (18.0)	397 (19.9)	771 (10.6)	0.135	<.0001	<.0001
Prior ICH	10 (0.6)	14 (0.7)	59 (0.8)	0.585	0.271	0.616
Prior GI bleeding	138 (7.7)	128 (6.4)	497 (6.9)	0.114	0.194	0.488
Renal disease	386 (21.6)	290 (14.5)	1,255 (17.3)	<0.001	<0.001	0.003
Liver disease	588 (32.9)	755 (37.8)	2,724 (37.5)	0.002	<0.001	0.828
Prior OAC user*	1,035 (58.0)	1,049 (52.5)	780 (10.8)	<0.001	<0.001	<0.001

Prior warfarin user	958 (53.6)	117 (5.9)	394 (5.4)	<0.001	<0.001	<0.001
Prior NOAC user	77 (4.3)	932 (46.7)	386 (5.3)	<0.001	0.083	<0.001
CHA_2DS_2-VASc score						
median (IQR)	4 (2 - 5)	4 (3 - 5)	3 (2 - 5)	<0.001	<0.001	<0.001
0	18 (1.0)	16 (0.8)	155 (2.1)	<0.001	<0.001	<0.001
1	160 (9.0)	116 (5.8)	826 (11.4)			
2	323 (18.1)	290 (14.5)	1,505 (20.7)			
3	354 (19.8)	376 (18.8)	1,428 (19.7)			
4	315 (17.6)	384 (19.2)	1,229 (16.9)			
5	301 (16.9)	341 (17.1)	1,033 (14.2)			
6	183 (10.3)	268 (13.4)	672 (9.3)			
7≤	132 (7.4)	206 (10.3)	408 (5.6)			
Modified HAS-BLED						
median (IQR)	3 (3 - 4)	3 (3 - 4)	3 (2 - 4)	0.034	<0.001	<0.001
1	72 (4.0)	35 (1.8)	391 (5.4)	0.011	<0.001	<0.001
2	286 (16.0)	272 (13.6)	1,532 (21.1)			
3	737 (41.3)	871 (43.6)	2,842 (39.2)			
4	450 (25.2)	575 (28.8)	1,811 (25.0)			
5	206 (11.5)	193 (9.7)	591 (8.1)			
6≤	35 (2.0)	51 (2.6)	89 (1.2)			
Concomitant medication						
Clopidogrel	1,701 (95.2)	1,889 (94.6)	5,065 (69.8)	0.365	<0.001	<0.001
Prasugrel or Ticagrelor	85 (4.8)	108 (5.4)	2,174 (30.0)	<0.001	<0.001	<0.001
NSAIDs	1,130 (63.3)	1,395 (69.9)	4,724 (65.1)	<0.001	0.146	<0.001
Statins	1,577 (88.3)	1,773 (88.8)	6,519 (89.8)	0.640	0.056	0.169

Loop diuretics	1,213 (67.9)	1,328 (66.5)	3,690 (50.9)	0.354	<.0001	<0.001
Beta-blockers	1,479 (82.8)	1,656 (82.9)	5,809 (80.1)	0.926	0.008	0.004
Calcium channel blockers	1,187 (66.5)	1,359 (68.1)	4,736 (65.3)	0.298	0.343	0.020
RAAS blockers	1,477 (82.7)	1,588 (79.5)	5,483 (75.6)	0.013	<0.001	<0.001
Proton pump inhibitors	961(53.8)	1,406 (70.4)	4,065 (56.0)	<0.001	0.091	<0.001

* within 1-year before the index PCI

ASD, absolute standardized difference; CABG, coronary artery bypass grafting; DAPT, dual-antiplatelet therapy; GI, gastrointestinal; ICH, intracranial hemorrhage; IQR, inter-quartile range; MI, myocardial infarction; NOAC, non-vitamin K oral anticoagulant; NSAIDs, non-steroidal anti-inflammatory drugs; OAC, oral anticoagulant; PCI, percutaneous coronary intervention; RAAS, renin-angiotensin-aldosterone system; SD, standard deviation; STE, systemic thromboembolism; TIA, transient ischemic attack; TT, triple therapy; VKA, vitamin K antagonist.

Values given as mean ± standard deviation, median (interquartile range), or number (percentage), unless otherwise indicated.
