# Supplementary material for: Comparison of early clinical outcomes between dual antiplatelet therapy and triple antithrombotic therapy in patients with atrial fibrillation undergoing percutaneous coronary intervention
Source: PLoS One. 2022 Feb 25;17(2):e0264538. doi: 10.1371/journal.pone.0264538 (PMC8880831; doi:10.1371/journal.pone.0264538)
Supplement: S3 Table — (PDF) [file pone.0264538.s003.pdf]

S3 Table. Clinical outcomes in dual-antiplatelet therapy group with potent $P2Y_{12}$ inhibitor

	Event (IR)			VKA-TT (ref) vs. DAPT	NOAC-TT (ref) vs. DAPT
	VKA-TT	NOAC-TT	DAPT	Adjusted HR (95% CI)	Adjusted HR (95% CI)
**Ischemic stroke**	33 (7.8)	17 (3.5)	16 (3.0)	0.54 (0.27–1.07)	1.29 (0.54–3.05)
**Myocardial infarction**	45 (10.6)	57 (12.0)	87 (16.9)	0.98 (0.66–1.45)	0.98 (0.67–1.44)
**All-cause death**	81 (18.8)	68 (14.1)	55 (10.4)	0.75 (0.51–1.11)	1.01 (0.67–1.55)
**Major bleeding**	31 (7.3)	36 (7.5)	23 (4.4)	0.64 (0.35–1.17)	0.69 (0.37–1.29)

CI, confidence interval; DAPT, dual antiplatelet therapy; HR, hazard ratios; IR, incidence rate; NOAC-TT, non-vitamin K oral anticoagulant-based triple therapy; OAC, oral anticoagulants; VKA-TT, vitamin K antagonist-based triple therapy
